# Supplementary material for: Light microscopy image segmentation using active contours driven by local image information for environmentally friendly fired-clay bricks design and characterization
Source: PLoS One. 2025 Aug 21;20(8):e0328270. doi: 10.1371/journal.pone.0328270 (PMC12370138; doi:10.1371/journal.pone.0328270)
Supplement: Attachment — Submitted filename: Editors and Reviewers Letter.docx [file pone.0328270.s001.docx]

**Dear Prof. André Gustavo de Sousa Galdino,**

**Academic Editor of the PLOS ONE Journal**

**Dear Reviewers,**

We sincerely thank you for considering our manuscript titled “Light Microscopy Image Segmentation Using Active Contours Driven by Local Image Information for Environmentally Friendly Fired-Clay Bricks Design and Characterization” for publication in **PLOS ONE**.

We are also grateful for the reviewers’ thoughtful evaluations, which significantly contributed to improving the manuscript. We have addressed all the comments and suggestions provided by the editors and reviewers, as detailed below. All corresponding modifications in the manuscript are highlighted in yellow.

**Editors:**

1. *Please ensure that your manuscript meets PLOS ONE's style requirements.*

Response (**R**): The PLOS ONE style templates have been used to format the manuscript in its current version.

2. *… please ensure that you provide the correct grant numbers for the awards you received for your study in the ‘Funding Information’ section.*

**R**: The Funding information section was updated:

3. *Please state what role the funders took in the study.*

**R**: In the current response to the editors and reviewers, we have added the following statement to clarify the role of the funder: “The funders had no role in study design, data collection and analysis, decision to publish, or preparation of the manuscript.”

4. *Please confirm at this time whether or not your submission contains all the raw data required to replicate the results of your study. Authors must share the “minimal data set” for their submission…*

**R**: The methodology and the mathematical background underlying the algorithm implementation are described throughout the manuscript. The uncertainty associated with the experimental setup is presented in Table 3, while the acquired images of the ceramic samples can be made available upon request.

5. *Please review your reference list to ensure that it is complete and correct.*

**R**: According to the reviewers’ suggestions, the reference list has been updated, and the rationale for including the suggested papers has been incorporated throughout the manuscript.

**Reviewer #1:**

*The manuscript presents an experimental study on fired clay bricks incorporating sewage sludges. The manuscript is not written well and is not well-organized. The novelty of the research is not clearly demonstrated. There are many relevant studies on the same topic on using sewage sludges in fired clay bricks (e.g., https://doi.org/10.1063/1.5002344;* [*https://doi.org/10.1016/j.rineng.2023.101708*](https://doi.org/10.1016/j.rineng.2023.101708)*).*

**R:** It is true that somehow the results were incomplete, and the contributions were not sufficiently underlined. Consequently, we respond to all the subsequent remarks, trying to address all the shortcomings in the new version of the manuscript. To begin with, we added the suggested references and underlined that our results fall within the range of high-quality bricks reported in the literature. Further on there is a paragraph from the new manuscript version (the changes were underlined in yellow within the manuscript):

“Aeslina et al. (2017) highlighted that incorporating 5% sewage sludge waste (SSW) in brick production yields bricks of acceptable quality, meeting the necessary standards for construction applications. Amir et al. (2024) utilized sewage sludge from Senggarang and Perwira in the fabrication of bricks intended for non-load-bearing applications, with results indicating that bricks incorporating Senggarang sludge outperformed those made with Perwira sludge. Moreover, Chindaprasirt et al. (2021) reported the production of fire clay bricks with 4% fly ash and glass cullet content which achieved a compressive strength of over 17.2 MPa, while maintaining low firing temperatures (i.e. 950 °C). As a result, the obtained compressive strength of 26 MPa in case of the N20 samples meets the criteria for the production of first-class bricks, in accordance with ASTM C62 standards for brick manufacturing, and falls within the range of high-quality bricks reported in the literature.”

*The authors claimed that incorporating the light microscopy image in analyzing the porosity of clay bricks in the manuscript. However, there is a noticeable difference in porosity measured between MIP and proposed image processing method, as shown in Table 2. The relevant statistical approach should also be incorporated to highlight the advantage of the proposed image processing method.*

**R:** In order to highlight the advantage of the proposed method compared with the classical level set approach, details regarding the comparative analysis conducted across all brick samples with various sludge content levels were added, focusing on both the accuracy of porosity estimation and the consistency of results. Thus, the following paragraphs were added related to the Table 2 measurements: “According to Table 2, the porosity evaluation of bricks incorporating increasing proportions of sewage sludge (5%, 10%, 12%, and 15%) was conducted using three techniques: classic level-set image segmentation (IAₜₕ), adaptive level-set image segmentation (IAₗₛ), and mercury intrusion porosimetry (mₚ). The results clearly reveal distinct trends in both porosity estimation and variability among these methods. Classic image processing consistently produces lower porosity values with moderate variability; for example, in the 5% sludge group, IAₜₕ yields a mean porosity of 4.1% with a standard deviation of 0.79%. This method, while straightforward, is limited by its reliance on fixed thresholds and its inability to accurately capture pore structures that lie outside the focal plane, making it less effective in characterizing complex or heterogeneous porosity. In contrast, the adaptive level-set method yields higher porosity values, reflecting its enhanced capability to detect fine-scale and out-of-focus pore features through dynamic boundary adaptation. For instance, in the same 5% sludge group, IAₗₛ reports a mean of 4.8%, closer to the mercury porosimetry value of 6.8%. Notably, IAₗₛ exhibits slightly higher variability than the classic method, with a standard deviation of 0.82% compared to 0.79%. This pattern persists across all sludge contents, suggesting that the adaptive segmentation introduces minor variability, possibly due to its dynamic boundary detection, but also enhances structural accuracy. Mercury intrusion porosimetry (mₚ), serving as the reference, consistently reports the highest porosity values with the lowest standard deviations. For example, at 15% sludge content, it yields a mean porosity of 14.2% with a low standard deviation of 0.59%. While adaptive level set segmentation introduces slightly more variability than the classic approach for the N17 to N19 samples, it provides porosity measurements that are significantly closer to those obtained via mercury intrusion porosimetry. This makes IAₗₛ a valuable compromise between non-destructive image-based techniques and the high-accuracy, but invasive, mercury intrusion-based method.”

*Language editing is recommended as there are multiple typos/grammar issues in the manuscript. The specific comments, questions, and suggestions are shown below:*

*1. The Introduction section is too long and lacks clarity and logical flow. It should be more directly connected to the research topic rather than presenting excessive general information (e.g., lines 160-171). Additionally, Section 1.2 should be removed and its key points integrated into a concise paragraph that effectively highlights the significance of this study.*

**R:** The initial version of the manuscript was intended to provide an introduction from both material characterization and image processing perspectives. However, addressing both areas in parallel may have resulted in an overly general overview. Consequently, in response to the reviewer’s suggestion, lines 160–171 and their corresponding bibliographic references were removed and replaced with concise introductory phrases. Additionally, the former section headings 1.1 and 1.2 were eliminated to ensure a more coherent and unified presentation of the introductory content.

*2. In section 2.1, the material characterization tests (e.g., XRD, TGA, XRF, etc.) for raw materials (clays and dry sewage sludge) should be performed.*

**R:** Although material characterization tests such as XRD, TGA, and XRF are indeed valuable for understanding the properties of raw materials like clays and sewage sludge, they were not included in the revised version of the manuscript, as their addition together with the already included analysis such as IR investigations and DTA-TG profiles would slightly deviate from the central focus on the image-based analysis procedure. Nonetheless, the authors acknowledge their relevance and consider them a valuable direction for complementary future studies to be published in material characterization journals.

*3. Line 291: the pressure unit is incorrect. There are lots of similar typos throughout the manuscript.*

**R:** The typo was corrected, and the manuscript was carefully revised.

*4. Line 295: Cooling rate is suggested to incorporate.*

**R:** Cooling rate information was added according to the following paragraph: “After the heating treatment, the samples were maintained in the oven and were slowly cooled to ambient temperature with no forced ventilation, with a cooling rate of approximately 40°C per hour, up to 200°C. The final cooling phase was carried out at a controlled rate of approximately 10 °C per hour, allowing the specimens to gradually reach ambient temperature without inducing thermal stress.”

*5. In section 2.2, the testing standards and references should be given. The authors did not present all tests performed in this study, e.g., water absorption.*

**R:** The water absorption standard values were specified according to the following paragraph:” The calculated mean for the measured water absorption coefficient of 13.7% falls below the maximum allowable limit of 17% specified by ASTM C62 for fired clay bricks”

*6. Line 544: wather?*

**R:** The underlined typos were corrected.

*7. Lines 590-603: Degree signs are misused.*

**R:** The correct degree signs are added along the manuscript.

*8. Although the 15% sewage sludge mixture achieved a compressive strength of 28 MPa (Table 1), exceeding the ASTM C62 requirements for brick strength, its water absorption increased to 13.7%. This rise in water absorption could significantly compromise the bricks' freeze-thaw durability. How did the authors address or mitigate this issue?*

**R:** Information regarding the water absorption coefficient and its compliance with ASTM standards were added within the revised version of the manuscript. Further on the added paragraph is cited: “The calculated mean for the measured water absorption coefficient of 13.7% falls below the maximum allowable limit of 17% specified by ASTM C62 for fired clay bricks intended for use in areas subject to severe weathering (Grade SW), indicating compliance with the standard and suitability for such environmental conditions.”

**Reviewer #2**:

The proposed image segmentation method, although innovative, may be complex for practical *applications. The reliance on active contours driven by local image information may require specialized knowledge and software, which could limit its accessibility for broader use in the industry;*

**R:** It is true that the proposed method introduces a certain level of complexity, particularly due to its reliance on active contours driven by local image information. However, this complexity is justified by the fact that the method addresses a fundamental and widely encountered challenge in optical microscopy image segmentation, namely the elimination of the defocus effect. By specifically tackling this issue, the method not only enhances the reliability and accuracy of segmentation results, which are critical for a broad range of applications in microscopy. In our case, it provides porosity measurements that are closer to those obtained via mercury intrusion porosimetry. In order to highlight the advantage of the proposed method compared with the classical level set approach, details regarding the comparative analysis conducted across all brick samples with various sludge content levels were added, focusing on both the accuracy of porosity estimation and the consistency of results.

*While thermal properties are enhanced, the addition of sewage sludge may reduce the mechanical strength of the bricks, posing challenges for structural applications, as the compressive strength may not meet all construction standards.*

***R:*** *To provide a deeper understanding of the material behavior and to enhance the practical relevance of the study, an in-depth analysis is introduced, aiming to establish a clear quantitative relationship between the bricks density, proportional with the porosity levels, and the corresponding compressive strength of the bricks. Figure 6 was introduced in the manuscript, which shows that the average compressive strength values of the specimens decreased progressively by approximately 10.6% (N18), 27.7% (N19), and 44.7% (N20) compared to the reference sample N17. Moreover, for underlining the limits of the proposed fired-clay bricks, the following paragraphs were added:*

*“Using the Gibson-Ashby scaling law (Gibson and Ashby, 1997), it is possible to predict to a certain extent the behavior of porous materials relative to the variation in relative density:*

*where P’ and ρ' refers to the properties of the compressive strength and density, whereas the lower indices s refers to the same properties, but for the reference sample with no enhanced porosity. The coefficient C2 is given by Gibson and Ashby for the compressive strength of metallic foams to lie typically between 0.1 and 1.0, with a theoretical exponent n=1.5. In our case the coefficient C2 is considered approximately 1, and the power exponent n was found to be 5.57. This indicates a more rapid decay of compressive strength with increasing porosity compared to metallic foams (n = 1.5). This observation is consistent with the susceptibility of ceramic-based materials to crack initiation and propagation as the porosity increases. Nevertheless, the power exponent falls within the interval of 3.0 to 6.0, corresponding to dense fired clay bricks (Gibson and Ashebey, 1997). This suggests that, despite the increased porosity introduced by sewage sludge incorporation, the mechanical performance of the obtained bricks remains within acceptable limits for high-quality fired clay products, confirming their suitability for practical construction applications.”*

*The manuscript does not address the potential variability in results due to different proportions of sewage sludge or other materials. A more thorough exploration of how varying these proportions affects thermal and mechanical properties could provide valuable insights for manufacturers*

**R:** In order to address the variability of the results, uncertainty associated with the experimental setup is detailed considering the camera precision and other factors according to the added paragraph: : “In our study, the uncertainty in the thermal properties measured using lock-in thermography…” Moreover, to highlight the advantage of the proposed method compared with the classical level set approach, details regarding the comparative analysis conducted across all brick samples with various sludge content levels were added, focusing on both the accuracy of porosity estimation and the consistency of results. Thus, the following paragraphs were added related to the Table 2 measurements: “According to Table 2, the porosity evaluation of bricks incorporating increasing proportions of sewage sludge (5%, 10%, 12%, and 15%) was conducted using three techniques:…”

*The study could be improved by including comparative analysis with existing methods for producing fired clay bricks. This would help contextualize the benefits of the proposed approach and demonstrate its advantages over traditional methods*

**R:** Within the added paragraphs from the revised manuscript version, we underlined that our results fall within the range of high-quality bricks reported in the literature. Further on there is a paragraph from the new manuscript version (the changes were underlined in yellow):

“Aeslina et al. (2017) highlighted that incorporating 5% sewage sludge waste (SSW) in brick production yields bricks of acceptable quality, meeting the necessary standards for construction applications. Amir et al. (2024) utilized sewage sludge from Senggarang and Perwira in the fabrication of bricks intended for non-load-bearing applications, with results indicating that bricks incorporating Senggarang sludge outperformed those made with Perwira sludge. Moreover, Chindaprasirt et al. (2021) reported the production of fire clay bricks with 4% fly ash and glass cullet content which achieved a compressive strength of over 17.2 MPa, while maintaining low firing temperatures (i.e. 950 °C). In comparison, the brick specimens proposed by Ebadi-Jamkhaneh et al. (2021) incorporating regular iron wire and cast-iron powder demonstrated even higher compressive strengths, further reinforcing the potential of these waste materials to produce environmentally sustainable and mechanically robust bricks. The results presented in Ahmadi et al. (2023) indicate that lower replacement ratios (e.g., 20% or 40%) for clay with water treatment sludge may offer a balance between environmental benefits and mechanical performance, but as the proportion of WTS increases, the negative effects on compressive strength become more significant. The best results were obtained for the lowest sludge ratio (i.e. 20%) where the compresive strength reaches 16 MPa. As a result of our study, the obtained compressive strength of 26 MPa in case of the N20 samples meets the criteria for the production of first-class bricks, in accordance with ASTM C62 standards for brick manufacturing, and falls within the range of high-quality bricks reported in the literature”

*Expanding the tests to include a wider range of conditions and material combinations would strengthen the conclusions and applicability of the research.*

Expanding the range of tested conditions and material combinations would definitely add more value and applicability of the research. In this regard, we would like to point out that, as mentioned in the manuscript, the use of alternative materials such as paper pulp has been reported in the literature. To further strengthen the conclusions and enhance the applicability of our research future work that can be included in a review article is envisaged, that will systematically compare various material solutions and broaden the context of our findings.

**Reviewer #3:**

*1- While the proposed active contour approach with local image information is novel, it is crucial to clearly justify why traditional approaches (such as standard Chan-Vese or machine learning techniques) are insufficient specifically for the porosity characterization of ceramic bricks. A detailed comparative analysis highlighting specific limitations would strengthen the methodological justification.*

To provide additional details regarding the importance of the proposed segmentation approach in accurately estimate the porosity levels of the brick samples under analysis, Table 2 has been updated to include, for all brick samples with varying sludge ratios, not only the mean porosity values but also the standard deviations for both the classical level set procedure (Chan-Vese) and the adaptive level set procedure proposed in this study. A comparative analysis is also included as detailed in the following paragraph extracted from the revised version of the manuscript:

“According to Table 2, the porosity evaluation of bricks incorporating increasing proportions of sewage sludge (5%, 10%, 12%, and 15%) was conducted using three techniques: classic level-set image segmentation (IAₜₕ), adaptive level-set image segmentation (IAₗₛ), and mercury intrusion porosimetry (mₚ). The results clearly reveal distinct trends in both porosity estimation and variability among these methods. Classic image processing consistently produces lower porosity values with moderate variability; for example, in the 5% sludge group, IAₜₕ yields a mean porosity of 4.1% with a standard deviation of 0.79%. This method, while straightforward, is limited by its reliance on fixed thresholds and its inability to accurately capture pore structures that lie outside the focal plane, making it less effective in characterizing complex or heterogeneous porosity. In contrast, the adaptive level-set method yields higher porosity values, reflecting its enhanced capability to detect fine-scale and out-of-focus pore features through dynamic boundary adaptation. For instance, in the same 5% sludge group, IAₗₛ reports a mean of 4.8%, closer to the mercury porosimetry value of 6.8%. Notably, IAₗₛ exhibits slightly higher variability than the classic method, with a standard deviation of 0.82% compared to 0.79%. This pattern persists across all sludge contents, suggesting that the adaptive segmentation introduces minor variability, possibly due to its dynamic boundary detection, but also enhances structural accuracy. Mercury intrusion porosimetry (mₚ), serving as the reference, consistently reports the highest porosity values with the lowest standard deviations. For example, at 15% sludge content, it yields a mean porosity of 14.2% with a low standard deviation of 0.59%. While adaptive level set segmentation introduces slightly more variability than the classic approach for the N17 to N19 samples, it provides porosity measurements that are significantly closer to those obtained via mercury intrusion porosimetry. This makes IAₗₛ a valuable compromise between non-destructive image-based techniques and the high-accuracy, but invasive, mercury intrusion based method.”

*2- The paper lacks an explicit quantitative validation method to rigorously demonstrate the accuracy of the proposed segmentation technique. Introducing performance metrics and clearly comparing the proposed approach with established methods would enhance credibility.*

We thank the reviewer for raising the point regarding the need for explicit quantitative validation of the proposed segmentation technique. In response, we have included more details regarding the comparative analysis between the classical image processing method (IAₜₕ), the proposed adaptive level-set method (IAₗₛ), and mercury intrusion porosimetry (mₚ), which serves as the reference standard. Quantitative results demonstrate that the classical threshold-based method (IAₜₕ) consistently yields lower porosity values and exhibits limited sensitivity to out-of-focus or fine-scale pore structures. For instance, in the 5% sludge group, IAₜₕ reports a mean porosity of 4.1% with a standard deviation of 0.79%, highlighting its moderate variability and limited capacity to detect complex porosity features. In contrast, the proposed adaptive level-set segmentation (IAₗₛ) produces porosity estimates that more accurately reflect the pore structures, including those outside the focal plane, thanks to its ability to dynamically adapt to boundary variations. In the same 5% sludge group, IAₗₛ yields a higher mean porosity of 4.8%, much closer to the 6.8% reported by mercury intrusion porosimetry (mₚ). Although IAₗₛ introduces a slightly higher variability (standard deviation of 0.82%), this is a reasonable trade-off for its improved structural accuracy. This trend is consistent across all tested sludge concentrations. For example, at 15% sludge content, mₚ reports 14.2% porosity with a low standard deviation of 0.59%, while IAₗₛ offers closer alignment with these values than IAₜₕ. These findings, included in the revised version of the manuscript, suggest that the proposed method not only enhances segmentation accuracy in terms of structural fidelity but also provides quantitative porosity measurements that better approximate than the classical image processing approach. In summary, the adaptive level-set method (IAₗₛ) strikes a practical balance between non-destructive image-based analysis and the high accuracy of mercury porosimetry. Its improved performance over classical methods validates its effectiveness for segmenting complex pore structures and substantiates the credibility of the proposed approach.

*3- The authors discussed addressing the defocus effect using the Laplace operator. However, no clear experimental results quantitatively demonstrate the effectiveness of this approach. Include detailed experimental data or quantitative metrics that explicitly demonstrate how effectively the proposed method handles defocus compared to existing methods.*

The authors acknowledge the reviewer’s observation regarding the lack of quantitative evidence for the effectiveness of the proposed method in addressing defocus. To address this, Figure 6 illustrates a detailed comparative analysis between porosity and defocused regions in image sections extracted from N20 samples, which include areas with large pores. The upper row (images a, b, and c) shows the original microstructural sections with visible pore features, while the corresponding lower row (a’, b’, c’) presents binary masks highlighting regions impacted by the defocus effect as detected via the Laplacian operator.

To provide quantitative validation, the following metrics were computed for each image: the percentage of the area affected by defocus, porosity estimated using the classical Chan–Vese active contour method, and the intersection between the defocus mask and the pore regions. In image 6.a, 8.74% of the area is defocused, porosity is 18.19%, and the intersection is 6.17%. For image 6.b, defocus covers 10.03% of the area, with a porosity of 21.42%, and an intersection of 8.46%. In image 6.c, 5.5% of the area is affected by defocus, porosity is 14.23%, and the intersection is 4.27%.

The average overlap between defocused regions and pore structures reaches approximately 75%, calculated as the mean ratio of intersecting area to defocused area across the three samples. This substantial overlap strongly supports the quantitative effectiveness that the Laplace-based defocus detection method reliably identifies regions spatially associated with pore structures. Consequently, the observed minor discrepancies between porosity values estimated by the classical Chan–Vese method and those obtained using the proposed adaptive active contour approach can be attributed to this strong spatial correlation. These results offer quantitative evidence supporting the effectiveness of the Laplace-based method in isolating defocus artifacts within porous regions.

*4- The manuscript indicates enhanced porosity leading to improved insulation properties; however, little attention is paid to the microstructural homogeneity of pores. Additional microstructural analyses, such as SEM images, would complement the existing microscopic data and confirm that pore formation remains homogeneous across samples.*

**R:** It is true that additional microstructural analysis would provide further insights and strengthen the study. However, we would like to mention that the primary focus of the current work is the development of a low-cost method for porosity estimation. While more detailed characterization could complement our findings, the pore histograms presented in the paper could offer an objective representation of the pore distributions across samples, underlining the benefits of the low-cost approach based on image processing tools for porosity estimation. Building on this foundation, our future work will expand to include more in-depth investigations, such as toxicological analyses of water sludge, XRD and XRF analyses, and, in line with your suggestion, SEM imaging to further characterize the microstructure.

5- Although the study demonstrates acceptable compressive strength for bricks incorporating up to 15% sewage sludge, a clear quantitative relationship or regression analysis linking porosity levels (quantitatively segmented via microscopy) to the observed mechanical strength would strengthen the practical relevance of the findings.

**R:** For strengthening the practical relevance of our study, an in-depth analysis is introduced, aiming to establish a clear quantitative relationship between the bricks density, proportional with the porosity levels, and the corresponding compressive strength of the bricks. Figure 6 was introduced in the manuscript, which shows that the average compressive strength values of the specimens decreased progressively by approximately 10.6% (N18), 27.7% (N19), and 44.7% (N20) compared to the reference sample N17. Moreover, the exponent n for the Gibson-Ashby scaling law used to predict the behavior of porous materials relative to the variation in relative density, was estimated to a value of n = 5.57, suggesting that, despite the increased porosity introduced by sewage sludge incorporation, the mechanical performance of the obtained bricks remains within acceptable limits for high-quality fired clay products, confirming their suitability for practical construction applications.

6- The thermogravimetric analysis provided valuable insights; however, the manuscript does not sufficiently correlate TGA mass loss results with observed changes in porosity or thermal conductivity values. Clearly establishing these relationships would add depth to the discussion on thermal decomposition dynamics in brick manufacturing.7- Stability and reproducibility of the segmentation results were not explicitly discussed. It would be beneficial if the authors performed a repeatability analysis by processing multiple images under slightly varied lighting or focus conditions to verify the robustness of the proposed segmentation method.

**R:** In order to include details regarding the stability and reproducibility of the segmentation results, Table 2 was modified by introducing, for all brick samples with different sludge ratios, besides the means, the standard deviation in case of porosity estimation with classical level set procedure and the adaptive level set procedure introduced by current work. According to Table 2, the porosity evaluation of bricks incorporating increasing proportions of sewage sludge (5%, 10%, 12%, and 15%) was conducted using three techniques: classic level-set image segmentation (IAₜₕ), adaptive level-set image segmentation (IAₗₛ), and mercury intrusion porosimetry (mₚ). The results clearly reveal distinct trends in both porosity estimation and variability among these methods. Classic image processing consistently produces lower porosity values with moderate variability; for example, in the 5% sludge group, IAₜₕ yields a mean porosity of 4.1% with a standard deviation of 0.79%. This method, while straightforward, is limited by its reliance on fixed thresholds and its inability to accurately capture pore structures that lie outside the focal plane, making it less effective in characterizing complex or heterogeneous porosity. In contrast, the adaptive level-set method yields higher porosity values, reflecting its enhanced capability to detect fine-scale and out-of-focus pore features through dynamic boundary adaptation. For instance, in the same 5% sludge group, IAₗₛ reports a mean of 4.8%, closer to the mercury porosimetry value of 6.8%. Notably, IAₗₛ exhibits slightly higher variability than the classic method, with a standard deviation of 0.82% compared to 0.79%. This pattern persists across all sludge contents, suggesting that the adaptive segmentation introduces minor variability, possibly due to its dynamic boundary detection, but also enhances structural accuracy. Mercury intrusion porosimetry (mₚ), serving as the reference, consistently reports the highest porosity values with the lowest standard deviations. For example, at 15% sludge content, it yields a mean porosity of 14.2% with a low standard deviation of 0.59%. While adaptive level set segmentation introduces slightly more variability than the classic approach for the N17 to N19 samples, it provides porosity measurements that are significantly closer to those obtained via mercury intrusion porosimetry. This makes IAₗₛ a valuable compromise between non-destructive image-based techniques and the high-accuracy, but invasive, mercury intrusion-based method.

*8- While MIP is cited as the comparative standard, the manuscript briefly acknowledges its limitations without clearly stating how this could affect comparative assessments. Clarify and explicitly state how these limitations were considered or compensated for during comparisons between MIP and the proposed image-based method.*

We appreciate the reviewer’s observation regarding the need to explicitly clarify how the limitations of mercury intrusion porosimetry (MIP) were considered during comparative analysis. Indeed, while MIP is widely regarded as a reliable technique for porosity quantification, it presents several known limitations that can impact the comparison with image-based methods. In the revised manuscript, we have emphasized that MIP tends to yield slightly higher porosity values than those estimated via adaptive image segmentation (IAₗₛ), primarily due to its ability to detect a wider range of pore sizes, including submicron and highly interconnected pores that might not be clearly resolved or accessible in microscopy images. Furthermore, MIP forces mercury into pores under high pressure, allowing detection of small or closed pores that may be partially occluded or affected by surface tension, thus not readily captured by optical methods. Conversely, our image-based approach predominantly captures larger, surface-connected pores visible in the microscopy plane. This discrepancy in pore size detection explains the observed differences between MIP and image segmentation porosity estimates To mitigate these differences and ensure meaningful comparisons, we analyzed the distribution of pores across different size classes (e.g., above and below 0.7 mm², as illustrated in Figure 4). This allowed us to better understand which pores are likely to contribute to each measurement method. Additionally, we examined how defocus effects influence segmentation in large-pore areas, as presented in Figure 5, and quantified the overlap between defocused and porous regions. The analysis revealed that approximately 75% of the defocused regions align with identified pores, reinforcing that the adaptive segmentation method—designed to compensate for defocus—more accurately captures structural porosity compared to classical approaches. While MIP remains the reference standard, these adjustments and validations enhance the reliability and comparability of our image-based technique, especially considering its non-invasive nature and practical applicability in microstructural analysis. We have now included this detailed explanation in the revised manuscript to explicitly clarify how the limitations of MIP were considered and accounted for during comparisons.

*9. For thermal property measurements using non-contact infrared lock-in thermography, it is important to state explicitly the measurement uncertainty and any assumptions in thermal diffusivity and conductivity calculations. Including these details will enhance the reliability of thermal characterization results.*

**R:** Considering our experimental setup, the uncertainty is estimated based on the following parameters: precision of the infrared camera, with a sensitivity of 20 mK, phase shift measurements with an uncertainty, and the laser power and frequency modulation uncertainty. The formula for uncertainty propagation in case multiple independent variables are involved is defined by the squared root of the sum of squares for each individual uncertainty. This resulted in the inclusion of the following paragraph within the final version of the manuscript: “In our study, the uncertainty in the thermal properties measured using lock-in thermography is quantified considering our experimental setup. The precision of the infrared camera, with a sensitivity of 20 mK, combined with phase shift measurements uncertainty of ±1%, and the uncertainty of the laser power and modulation frequency of ±1%, lead to an overall minimum uncertainty of ±2% for the thermal parameters. Since the uncertainties propagate differently for each parameter, the maximum uncertainty will vary depending on which parameter has the greatest impact. Considering also the contributions from the signal-to-noise ratio (SNR) together with an uncertainty increase that may appear due to the camera precision, the maximum uncertainty in the determination of thermal effusivity, conductivity, and diffusivity reaches up to approximately 5%.”

10- Given the scope and content of this paper, it may benefit from considering the following related works:
<https://doi.org/10.3390/su15129389>, <https://doi.org/10.1088/1755-1315/899/1/012042>, <https://doi.org/10.1061/(ASCE)MT.1943-5533.0003737>

**R**: The works mentioned were included, by comparing the reported compressive strength with the one obtained within our study. The corresponding paragraph is listed as follows: In comparison, the brick specimens proposed by Ebadi-Jamkhaneh et al. (2021) incorporating regular iron wire and cast-iron powder demonstrated even higher compressive strengths, further reinforcing the potential of these waste materials to produce environmentally sustainable and mechanically robust bricks. The results presented in Ahmadi et al. (2023) indicate that lower replacement ratios (e.g., 20% or 40%) for clay with water treatment sludge may offer a balance between environmental benefits and mechanical performance, but as the proportion of WTS increases, the negative effects on compressive strength become more significant. The best results were obtained for the lowest sludge ratio (i.e. 20%) where the compressive strength reaches 16 MPa. As a result of our study, the obtained compressive strength of 26 MPa in case of the N20 samples meets the criteria for the production of first-class bricks, in accordance with ASTM C62 standards for brick manufacturing, and falls within the range of high-quality bricks reported in the literature.

Thank you for evaluating our manuscript,

Looking forward for your response,

Kind Regards,

Corresponding Author,

Belean Ioan Bogdan
